# Supplementary material for: Insight into the RssB-Mediated Recognition and Delivery of σs to the AAA+ Protease, ClpXP
Source: Biomolecules. 2020 Apr 16;10(4):615. doi: 10.3390/biom10040615 (PMC7226468; doi:10.3390/biom10040615)
Supplement: Supplementary file 1 [file biomolecules-10-00615-s001.pdf]

Insight into the RssB-Mediated Recognition and Delivery of σ^s to the AAA+ Protease, ClpXP

Dimce Micevski[1], Kornelius Zeth[2,3], Terrence D. Mulhern[4], Verena J. Schuenemann[2,†], Jessica E. Zammit[1], Kaye N. Truscott[1,*] and David A. Dougan[1,*]

1 Department of Biochemistry and Genetics, La Trobe Institute for Molecular Science, La Trobe University, Melbourne, Victoria, 3086, Australia jmicevski85@hotmail.com (D.M.); 16111322@students.latrobe.edu.au (J.E.Z.);
2 Department of Protein Evolution, Max-Planck-Institute for Developmental Biology, Tübingen D-72076, Germany verena.schuenemann@iem.uzh.ch (V.J.S.)
3 Department of Science and Environment, Roskilde University, Roskilde DK-4000, Denmark kzeth@ruc.dk (K.Z.)
4 Department of Biochemistry and Molecular Biology, The University of Melbourne, Parkville, Victoria 3010, Australia, tmulhern@unimelb.edu.au (T.D.M.)
* Correspondence: k.truscott@latrobe.edu.au (K.N.T.); d.dougan@latrobe.edu.au (D.A.D.); Tel.: +61-3-9479-5245 (K.N.T.); +61-3-9479-3276 (D.A.D.)
† Present address: Institute of Evolutionary Medicine, University of Zurich, Zurich, Switzerland.

Table S1. Oligonucleotide primers used in this study

Primer	*Oligonucleotide sequence (5′-> 3′)*	*Gene*	*Features*
5Ub-sigS	GACTCTCCGCGGTGGAAGTCAGAATACGCTGAAAGTTC	*rpoS*	*Sac* II *restriction site for cloning into pHUE*
3sigS_hind	TGGTCGAAGCTTACTCGCGGAACAGCGC	*rpoS*	*Hind* III *restriction site for cloning into pHUE*
sac2_dN_rpoS	GCGCCTCCGCGGTGGAACACAGCGTGTGTTGGAC	*rpoS*	*Sac* II *restriction site for cloning into pHUE*
RssB_bam	GACTCTGGATCCATGACGCAGCCATTGGTCGG	*rssB*	*Bam* HI *restriction site for cloning into pHUE*
RssB_hind	TATGATAAGCTTCATTCTGCAGACAACATCAAG	*rssB*	*Hind* III *restriction site for cloning into pHUE*
RssB_N1	CCCAGCATGTAAGGATCCCGCGTTGAGGAAGAGGAAAGG	*rssB*, *rssB*$_C$	*Introduce Bam* HI *restriction site to create RssB*$_N$ *in pHUE or pET32 and RssB*$_C$ *in pHUE*
RssB_N2	CCTCAACGCGGGATCCTTACATGCTGGGATAGAGACAGG	*rssB*, *rssB*$_C$	*Introduce Bam* HI *restriction site to create RssB*$_N$ *in pHUE or pET32 and RssB*$_C$ *in pHUE*
Nco_rssBc	TAGATCCCATGGTGTTTAATTCGCGCGTTG	*rssB*$_C$	*Introduce Nco* I *restriction site to create RssB*$_C$ *in pET32*
rssB_stop_xho	CATCAGCTCGAGTCATTCTGCAGACAACATC	*rssB*$_C$	*Introduce Xho* I *restriction site to create RssB*$_C$ *in pET32*
D58E_1	GATGATATGTGAAATCGCGATGCCACGAATG	*rssB*	*Introduce D58E mutation, removes Eco* RV *restriction site*
D58E_2	CATCGCGATTTCACATATCATCAGGTCTGGAG	*rssB*	*Introduce D58E mutation, removes Eco* RV *restriction site*
D58K_1	GATGATATGTAAAATCGCGATGCCACGAATG	*rssB*	*Introduce D58K mutation, removes Eco* RV *restriction site*
D58K_2	CATCGCGATTTTACATATCATCAGGTCTGGAG	*rssB*	*Introduce D58K mutation, removes Eco* RV *restriction site*
K108D_1	GTTTTGCTGGATCCAGTTAAAGATCTGAATC	*rssB*	*Introduce K108R mutation, introduce Bam* HI *restriction site*
K108D_2	CTTTAACTGGATCCAGCAAAACATCTTCAACG	*rssB*	*Introduce K108R mutation, introduce Bam* HI *restriction site*
K108R_1	GTTTTGCTGCGTCCAGTTAAAGATCTGAATC	*rssB*	*Introduce K108R mutation, removes Bam* HI *restriction site*
K108R_2	CTTTAACTGGACGCAGCAAAACATCTTCAACG	*rssB*	*Introduce K108R mutation, removes Bam* HI *restriction site*
RE_AA_1	GAATCGCTTGGCCGCCATGGTTTTTGCCTGTCTCTATCCCAG	*rssB*	*Introduce RE>AA mutation, introduce Nco* I *restriction site*
RE_AA_2	CAGGCAAAAACCATGGCGGCCAAGCGATTCAGATCTTTAAC	*rssB*	*Introduce RE>AA mutation, introduce Nco* I *restriction site*
R117A_1	GAATCGCTTGGCCGAGATGGTTTTTGCCTGTCTCTATCCCAG	*rssB*	*Introduce R117A mutation, remove Not* I *restriction site*
R117A_2	CAGGCAAAAACCATCTCGGCCAAGCGATTCAGATCTTTAAC	*rssB*	*Introduce R117A mutation, remove Not* I *restriction site*

[1] restriction sites for cloning and/or screening are underlined

Table S2. Plasmids used in this study

**Name**	**Description**		**Source**
	**gene**	**plasmid**	
pDT1010	rssB	pET32	Kind gift from Prof. Hengge<br>Bouche et al., (1998) Mol. Micro. 27, 787-795
pDT1528	rpoS	pHUE	Amplified *rpoS* using 5Ub-sigS and sigS_hind3, digested with *Sac* II and *Hind* III and cloned into pHUE
pDT2102	rssB	pHUE	Amplified *rssB* using RssB_bam and RssB_hind, digested with *Bam* HI and *Hind* III and cloned into pHUE
pDT2134	$rssB_N$	pHUE	Quick change mutagenesis using pDT2102 and primers RssB_N1 and RssB_N2
pDT2149	$rssB_C$	pHUE	Quick change mutagenesis using pDT2102 and primers RssB_N1 and RssB_N2, digestion with *Bam* HI to remove the fragment coding for $rssB_N$ followed by ligation of digested plasmid
pDT2102	rssB	pHUE	Amplified rssB using RssB_bam and RssB_hind, digested with *Bam* HI and *Hind* III and cloned into pHUE
pDT2470	rssB-D58K	pET32	Quick change mutagenesis using pDT1010 and primers D58K_1 and D58K_2
pDT2478	rssB-K108D	pET32	Quick change mutagenesis using pDT1010 and primers K108D_1 and K108D_2
pDT2499	rssB-RE>AA	pET32	Quick change mutagenesis using pDT2478 and primers RE_AA_1 and RE_AA_2
pDT2503	rssB-D58E	pET32	Quick change mutagenesis using pDT1010 and primers D58E_1 and D58E_2
pDT2520	rssB-R117A	pET32	Quick change mutagenesis using pDT1010 and primers R117A_1 and R117A_2
pDT2542	rssB-K108R	pET32	Quick change mutagenesis using pDT2478 and primers K108R_1 and K108R_2
pDT2854	rpoS-ΔN	pHUE	Amplified rpoS using sac2_dN_rpoS and sigS_hind3, digested with *Sac* II and *Hind* III and cloned into pHUE

Table S3 RssB Peptide library spot sequences

Spot	aa	sequence
1	1	MTQPLVGKQILIV
2	4	PLVGKQILIVEDE
3	7	GKQILIVEDEQVF
4	10	ILIVEDEQVFRSL
5	13	VEDEQVFRSLLDS
6	16	EQVFRSLLDSWFS
7	19	FRSLLDSWFSSLG
8	22	LLDSWFSSLGATT
9	25	SWFSSLGATTVLA
10	28	SSLGATTVLAADG
11	31	GATTVLAADGVDA
12	34	TVLAADGVDALEL
13	37	AADGVDALELLGG
14	40	GVDALELLGGFTP
15	43	ALELLGGFTPDLM
16	46	LLGGFTPDLMICD
17	49	GFTPDLMICDIAM
18	52	PDLMICDIAMPRM
19	55	MICDIAMPRMNGL
20	58	DIAMPRMNGLKLL
21	61	MPRMNGLKLLEHI
22	64	MNGLKLLEHIRNR
23	67	LKLLEHIRNRGDQ
24	70	LEHIRNRGDQTPV
25	73	IRNRGDQTPVLVI
26	76	RGDQTPVLVISAT
27	79	QTPVLVISATENM
28	82	VLVISATENMADI
29	85	ISATENMADIAKA
30	88	TENMADIAKALRL
31	91	MADIAKALRLGVE
32	94	IAKALRLGVEDVL
33	97	ALRLGVEDVLLKP
34	100	LGVEDVLLKPVKD
35	103	EDVLLKPVKDLNR
36	106	LLKPVKDLNRLRE
37	109	PVKDLNRLREMVF
38	112	DLNRLREMVFACL
39	115	RLREMVFACLYPS
40	118	EMVFACLYPSMFN
41	121	FACLYPSMFNSRV
42	124	LYPSMFNSRVEEE
43	127	SMFNSRVEEEERL
44	130	NSRVEEEERLFRD
45	133	VEEEERLFRDWDA
46	136	EERLFRDWDAMVD
47	139	LFRDWDAMVDNPA
48	142	DWDAMVDNPAAAA
49	145	AMVDNPAAAAKLL
50	148	DNPAAAAKLLQEL
51	151	AAAAKLLQELQPP
52	154	AKLLQELQPPVQQ
53	157	LQELQPPVQQVIS
54	160	LQPPVQQVISHCR
55	163	PVQQVISHCRVNY
56	166	QVISHCRVNYRQL
57	169	SHCRVNYRQLVAA
58	172	RVNYRQLVAADKP
59	175	YRQLVAADKPGLV
60	178	LVAADKPGLVLDI
61	181	ADKPGLVLDIAAL
62	184	PGLVLDIAALSEN
63	187	VLDIAALSENDLA
64	190	IAALSENDLAFYC
65	193	LSENDLAFYCLDV
66	196	NDLAFYCLDVTRA
67	199	AFYCLDVTRAGHN
68	202	CLDVTRAGHNGVL
69	205	VTRAGHNGVLAAL
70	208	AGHNGVLAALLLR
71	211	NGVLAALLLRALF
72	214	LAALLLRALFNGL
73	217	LLLRALFNGLLQE
74	220	RALFNGLLQEQLA
75	223	FNGLLQEQLAHQN
76	226	LLQEQLAHQNQRL
77	229	EQLAHQNQRLPEL
78	232	AHQNQRLPELGAL
79	235	NQRLPELGALLKQ
80	238	LPELGALLKQVNH
81	241	LGALLKQVNHLLR
82	244	LLKQVNHLLRQAN
83	247	QVNHLLRQANLPG
84	250	HLLRQANLPGQFP
85	253	RQANLPGQFPLLV
86	256	NLPGQFPLLVGYY
87	259	GQFPLLVGYYHRE
88	262	PLLVGYYHRELKN
89	265	VGYYHRELKNLIL
90	268	YHRELKNLILVSA
91	271	ELKNLILVSAGLN
92	274	NLILVSAGLNATL
93	277	LVSAGLNATLNTG
94	280	AGLNATLNTGEHQ
95	283	NATLNTGEHQVQI
96	286	LNTGEHQVQISNG
97	289	GEHQVQISNGVPL
98	292	QVQISNGVPLGTL
99	295	ISNGVPLGTLGNA
100	298	GVPLGTLGNAYLN
101	301	LGTLGNAYLNQLS
102	304	LGNAYLNQLSQRC
103	307	AYLNQLSQRCDAW
104	310	NQLSQRCDAWQCQ
105	313	SQRCDAWQCQIWG
106	316	CDAWQCQIWGTGG
107	319	WQCQIWGTGGRLR
108	322	QIWGTGGRLRLML
109	325	GTGGRLRLMLSAE

Table S4. Data collection and refinement statistics of $RssB_N$ and $RssB_C$.

Data collection/Refinement	$RssB_N$	$RssB_C$
**Data collection**		
Space group	$P3_2$	$P6_522$
Cell dimension		
a, *b*, *c* (Å)	45.13, 45.13, 95.30	56.24, 56.24, 294.46
α, β, γ (°)	90, 90, 120	90, 90, 120
Resolution (Å)	50-2.06 (2.17-2.06)	50-2.0 (2.05-2.0)
R_{sym} or R_{merge}	0.05 (0.88)	0.10 (1.32)
CC* in outermost shell	40.0	85.4
$I / \sigma I$	8.86 (1.03)	25.23 (2.84)
Completeness (%)	96.7 (96.9)	91.8 (99.9)
Redundancy	2.2 (2.2)	20.1 (20.0)
**Refinement**		
Program	PHENIX	PHENIX
Resolution (Å)	50-2.06 (2.22-2.06)	50-2.0 (2.1-2.0)
No. reflections	13508	18220
R_{work} / R_{free}	0.22/0.24	0.20/0.23
No. atoms (all)	1979	1789
Protein	1946	1652
Water	33	137
B-factors		
Protein	61.9	42.1
Water	72.5	47.8
R.m.s. deviations		
Bond lengths (Å)	0.005	0.004
Bond angles (°)	0.964	0.77
**Ramachandran statistics**		
Residues in favored region No (%)	97.6	96.6
Residues in allowed region No (%)	2.4	3.4
Residues in outlier region No (%)	0	0
**PDB-entry**		

**Values in parentheses are for highest-resolution shell*

Table S5. Preliminary SAXS modelling data for σ^s, RssB and the RssB/σ^s complex.

Protein	MW (kDa)	R_g (Å)	R_g Err. (Å)	D_{max} (Å)
σ^s	44895	31.80	0.66	105
Trx-$RssB_{R117A}$	49064	30.50	0.84	107
Trx-$RssB_{R117A}$/σ^s	91446	37.80	0.6	132

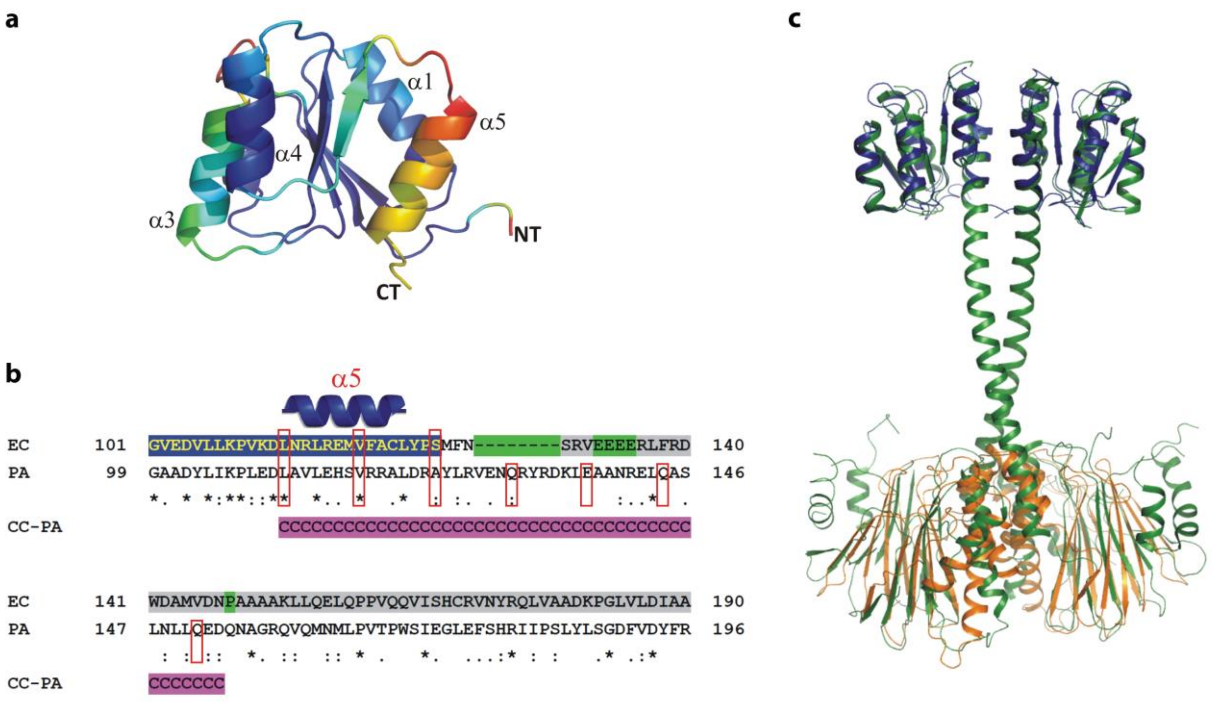

Figure S1 – Structural comparison of ^{Ec}RssB$_N$ and ^{Ec}RssB$_C$ with full-length ^{Pa}RssB

(a) Ribbon diagram of RssB$_N$ illustrating B-factors, which are colored from low (blue) to high (red). **(b)** Primary sequence alignment of the coiled coil (CC) region of ^{Pa}**RssB** (PA) in comparison to the equivalent region in ^{Ec}**RssB** (EC). Boxed residues highlight critical heptad repeat residues. **(c)** Superposition of ^{Ec}RssB$_C$ shown in orange and ^{Ec}RssB$_N$ shown in dark blue with the structure of *P. aeruginosa* RssB (PDB-entry: 3F7A) shown in green.

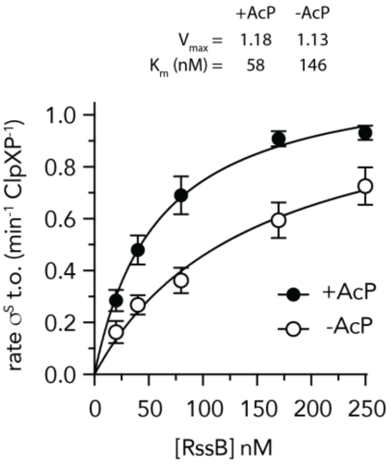

Figure S2 – Phosphorylation of RssB (by AcP) alters the apparent Km of σ^s turnover

The RssB-mediated turnover of σ^s by ClpXP was monitored in the presence of increasing concentrations of RssB (17 – 250 nM) with and without the addition of AcP (20 mM) . The rate of σ^s turnover (t.o.) was calculated by quantitation of at least three independent experiments (n>3). Error bars represent the standard error of the mean (S.E.M.).

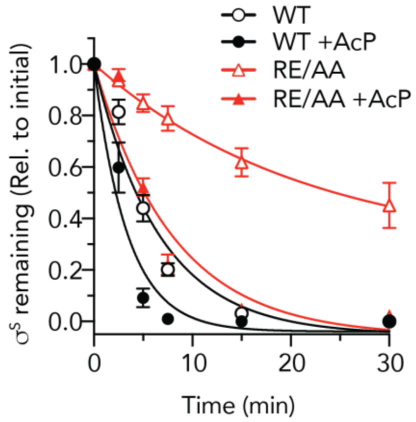

Figure S3 Quantitation of ClpXP-mediated degradation of σ^s by wild type RssB (black symbols) and RE/AA (red symbols) in the presence (filled symbols) or absence (open symbols) of AcP. Error bars represent S.E.M. of at least three independent experiments (n > 3).

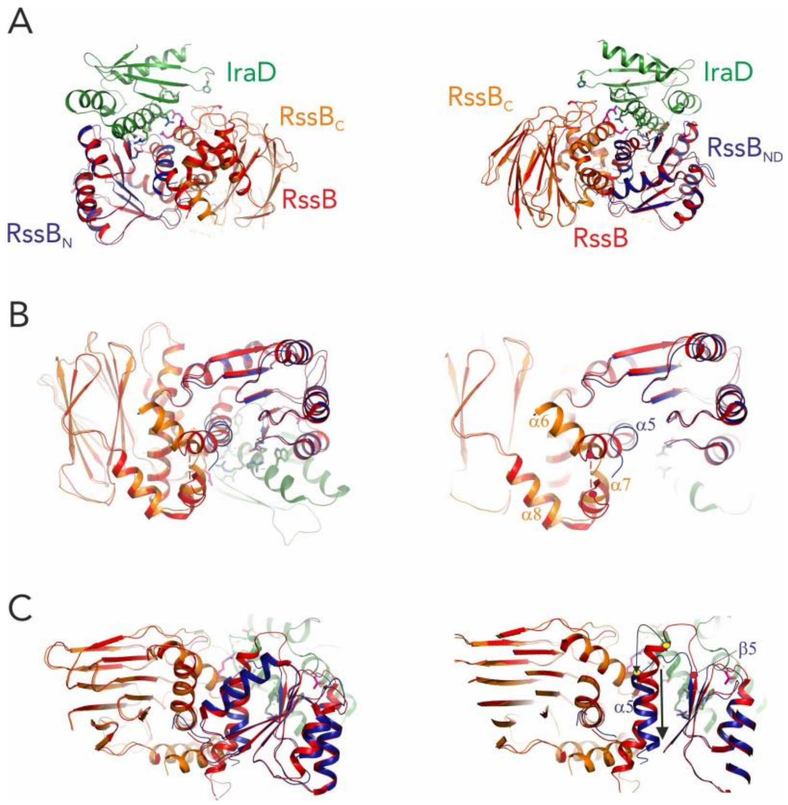

Figure S4. Superposition of the N- and C-terminal domain of RssB onto the complex of full-length RssB/IraD. The three structure are shown in ribbon representation. The RssB-IraD complex (PDB: 6OD1) is color coded red for RssB and green for IraD, while the N-domain is color coded blue and the C-terminal domain is in orange. The main changes are at the domain interface with a significant change in α5 helix of the N-terminal domain.
